# SARS-CoV-2 Infection and Lung Cancer: Potential Therapeutic Modalities

**DOI:** 10.3390/cancers12082186

**Published:** 2020-08-05

**Authors:** Ishita Gupta, Balsam Rizeq, Eyad Elkord, Semir Vranic, Ala-Eddin Al Moustafa

**Affiliations:** 1 College of Medicine, QU Health, Qatar University, 2713 Doha, Qatar; ishugupta28@gmail.com (I.G.); br1512683@student.qu.edu.qa (B.R.); semir.vranic@gmail.com (S.V.); 2 Biomedical Research Center, Qatar University, 2713 Doha, Qatar; 3 Qatar Biomedical Research Institute & 4Hamad Bin Khalifa University, 34110 Doha, Qatar; eelkord@hbku.edu.qa; 4 Biomedical Research Center, School of Science, Engineering and Environment, University of Salford, Manchester M5 4WT, UK

**Keywords:** COVID-19, Coronavirus, SARS-CoV-2, lung cancer, ACE-2, TMPRSS2, inhibitors

## Abstract

Human coronaviruses, especially SARS-CoV-2, are emerging pandemic infectious diseases with high morbidity and mortality in certain group of patients. In general, SARS-CoV-2 causes symptoms ranging from the common cold to severe conditions accompanied by lung injury, acute respiratory distress syndrome in addition to other organs’ destruction. The main impact upon SARS-CoV-2 infection is damage to alveolar and acute respiratory failure. Thus, lung cancer patients are identified as a particularly high-risk group for SARS-CoV-2 infection and its complications. On the other hand, it has been reported that SARS-CoV-2 spike (S) protein binds to angiotensin-converting enzyme 2 (ACE-2), that promotes cellular entry of this virus in concert with host proteases, principally transmembrane serine protease 2 (TMPRSS2). Today, there are no vaccines and/or effective drugs against the SARS-CoV-2 coronavirus. Thus, manipulation of key entry genes of this virus especially in lung cancer patients could be one of the best approaches to manage SARS-CoV-2 infection in this group of patients. We herein provide a comprehensive and up-to-date overview of the role of *ACE-2* and *TMPRSS2* genes, as key entry elements as well as therapeutic targets for SARS-CoV-2 infection, which can help to better understand the applications and capacities of various remedial approaches for infected individuals, especially those with lung cancer.

## 1. Introduction

Lung cancer is the second most common malignancy in both men and women and accounts for 75–80% of cancer-related deaths, making it the leading cause of mortality worldwide [1]. Lung cancer is a heterogeneous disease and can occur in various sites along the bronchial tree; based upon the anatomical location different symptoms are present and classified into four types [2]. Tumors that rise in the main bronchi and spreads to the carina are squamous cell lung cancers (SCC) and accounts for 25–30% of all lung cancers, while those arising in the peripheral bronchi exhibit glandular morphology and are classified as adenocarcinomas and account for 40% of all lung cancers [2]. Furthermore, tumors which lack the classic glandular or squamous morphology but exhibit neuroendocrine differentiation are classified as small-cell lung carcinomas (SCLC) and constitute ~15–20% of all lung carcinomas [2]. The remaining subtype without any of the afore-mentioned morphologies is called large cell carcinomas, some of which may also have neuroendocrine differentiation (Large cell neuroendocrine carcinomas). SCC, adenocarcinomas and large cell carcinomas are usually categorized as non-small cell lung carcinomas (NSCLC) as they have different pathological, molecular genetics and clinical characteristics in comparison with SCLC. It is believed, that both genetic, as in family history and polymorphisms, and environmental risk factors are responsible for lung cancer; smoking being the major risk factor for this disease [3,4]. Several recent studies have highlighted infectious agents such as bacteria [5,6,7] and viruses [8,9,10,11] as high-risk factors of the disease.

It is projected that at least 20% of all human cancers are attributed to microorganisms’ infections including viral [12]; viruses are linked with the onset of both solid and hematological malignancies in humans [13]. Some of the frequently linked oncoviruses with lung cancer include Epstein Barr virus (EBV), hepatitis viruses B and C (HBV and HCV), and human papillomaviruses (HPVs) [8,9,11]. However, their causal relationship to lung cancer has not been established yet. Some other viruses such as coronaviruses have been implicated in various respiratory diseases including pneumonia, upper respiratory tract infections, severe acute respiratory syndrome (SARS) and Middle-East respiratory syndrome (MERS) in addition to the recent coronavirus disease 2019 (COVID-19) [14,15,16]. However, the role of the severe acute respiratory syndrome coronavirus 2 (SARS-CoV-2) in lung cancer patients is still unclear. This review will focus on the mechanisms linking SARS-CoV-2 to cancer, especially lung cancer and the potential clinical relevance of the various inhibitors of SARS-CoV-2.

## 2. SARS-CoV-2 and Its Key Entry Genes

During December 2019 in Wuhan, China, several patients were diagnosed with atypical pneumonia and these infections were associated with a zoonotic origin [17,18]. The investigations determined β-coronavirus as the causative agent leading to the identification of SARS-CoV-2 [19]. Genetic analysis listed SARS-CoV-2 in the genus Betacoronavirus and subgenus Sarbecovirus (lineage B); confirming its origin to the bat coronavirus (BatCoV RaTG13) [19]; it is supposedly linked to the bat and pangolin coronavirus as well as SARS-CoV [19,20]. Further analysis showed only a single amino acid difference between SARS-CoV and the pangolin Coronavirus, suggesting a potential transitional host [21].

The coronavirus S protein consists of two functional domains; S1 is the receptor-binding domain while, S2 contains functional elements and plays a role in membrane fusion [22]. One of the distinctive features of the coronavirus S protein is that it consists of more than one proteolytic cleavage site; the first known cleavage site is present at the S1/S2 boundary, while the other is in the S2 upstream of the putative fusion peptide [23]. Once the spike glycoprotein cleaves, the S1 and S2 domains remain linked non-covalently; this association remains after cleavage of spike glycoprotein, then the S1 domain sheds itself from the S2 stalk domain of the protein [23,24,25].

The pathogenic mechanisms of SARS-CoV-2 are still poorly understood. At the cellular level, the viral particles enter into the host epithelial cell of the respiratory tract via cellular recognition using a metallopeptidase. Similar to SARS-CoV, SARS-CoV-2 uses angiotensin converting enzyme II (ACE-2) for viral entry; ACE-2 is a cellular receptor and plays a role in the breakdown of angiotensin II to regulate the renin–angiotensin system (RAS) [26]. The viral S protein combines with the ACE-2 receptor to enhance cellular membrane fusion and endocytosis; this process is dependent on S protein and is modulated by type II transmembrane serine proteases (TTSPs) [27,28]. TTSPs, such as TMPRSS2 and TMPRSS11D, play critical roles in spike protein cleavage and activate SARS-CoV permitting entry into the host by membrane fusion [29]. This indicates that TTSPs might play a principle role in SARS-CoV-2 spread and infections. On the other hand, additional proteases are indicated in the priming of SARS-CoV-2 but not SARS-CoV; one potential protease is FURIN [30]. Unlike SARS-CoV, SARS-CoV-2 protein consists of four redundant FURIN cut sites (PRRA motif) [30]. The FURIN protease allows effective cleavage of the SARS-CoV-2 protein [30], which upon receptor binding enhances viral entry into the host cell [31,32].

The renin–angiotensin system (RAS) is known as one of the major regulatory systems for maintaining blood pressure and body fluid homoeostasis [33]. ACE converts the inactive peptide hormone angiotensin I (Ang-I) to an active octapeptide, angiotensin II (Ang-II) [34,35]. Ang-II mediates its biological effects by binding to two receptors, which belong to the G-protein coupled receptor family, the angiotensin type I and type II receptors (AT1R and AT2R) [36]. While both receptors are responsible for signal transduction, they result in opposing effects, with AT1R activation leading to increased cell proliferation and vasoconstriction of blood vessels, while AT2R activation leads to decreased cell proliferation and vasodilation [37]. Binding of Ang-II to specific receptors activates a number of different events in various tissues and cell types [38]. In 2000, a homologue of ACE (ACE-2) was identified in humans [39]. ACE-2 is a type 1 integral membrane zinc-metalloprotease glycoprotein present in many cardiovascular-relevant organs, such as heart, kidneys, blood vessels, and lungs [40,41,42]. ACE-2 is also found in vascular smooth muscle cells, gastrointestinal tract, liver, pancreas, retina, central nervous system, bone marrow, and lymphoid tissues although at lower levels when compared to heart or kidneys (reviewed in [43,44]). ACE-2 is a key-regulator within the RAS and counteracts with ACE in two ways: Either it metabolizes the vasoconstrictive and pro-inflammatory Ang (1-8) directly to generate the vasodilatory and anti-proliferative Ang (1-7), a counter-regulatory enzyme to ACE, or it competes for Ang (1-10) with ACE to form Ang (1-9), a precursor of Ang (1-7) [45]. Consequently, recognition of the two pathways of Ang (1-7) production via ACE-2, has opened a new avenue to understand cardiovascular and lung physiology as well as provide new potential targets and therapeutic agents [39,46]. This was demonstrated by Mas knockout mice that have no antidiuretic action of Ang (1-7) after an acute water load and whose aortas lose their angiotensin (1-7)-induced relaxation response [47]. Ang (1-7) has the most promising therapeutic target due to its opposing effects to Ang-II showing an anti-hypertensive, anti-hypertrophic, anti-fibrotic, and anti-thrombotic properties [48]. However, therapeutic application of Ang (1-7) is limited due to its short half-life and rapid turnover [49]. Hence, another approach of raising levels of Ang (1-7) is to increase the catalytic activity or amount of ACE-2. Undoubtedly RAS is an intricate signaling system with numerous possible ligands, receptors and ligand/receptor interactions that need to be investigated extensively.

Type II transmembrane serine proteases (TTSPs) is the largest group of membrane-anchored serine proteases [50]. To date, nineteen human TTSps have been identified. TTSPs are classified into four subfamilies including Hepsin/TMPRSS, Matriptase, HAT/DESC, and Corin [51]. Several different isoforms exist in humans and rodents [51]; however, there are two non-mammalian TTSPs, Drosophila stubble-stubbloid (st-sb) and corin [52,53]. The TMPRSS2 gene, also known as epitheliasin belongs to the serine protease family that is located on human chromosome 21q22.3 and encodes a polypeptide of 492 amino acids [54,55]. TMPRSS2 is 70-kDa and is almost 44kb in length with 14 exons that are involved in various cellular processes including digestion, tissue remodeling, fertility, blood coagulation, inflammatory responses, tumor cell invasion, and apoptosis [54,55]. While in-vitro translated TMPRSS2 protein is present as a zymogen with a molecular mass of around 54 kDa [56], the intrinsic and recombinant human TMPRSS2 proteins undergo N-linked glycosylation and have a higher molecular mass of approximately 60–70 kDa [56,57]. TMPRSS2 consists of five domains: Type II transmembrane, low-density lipoprotein (receptor class A), scavenger receptor cysteine-rich, protease and cytoplasmic domains [54]; however, the normal physiological role of TMPRSS2 is still unknown. Studies using the Xenopus oocyte expression system revealed that TMPRSS2 expression reduced both the epithelial sodium channel and protein levels [58].

## 3. Role of ACE-2 and TMPRSS2 in Lung Disease/COVID-19

ACE-2 expression is virtually present in all tissues with relatively higher expression in respiratory epithelial cells, arteries, arterioles, and venules in the heart and kidney, alveolar cells type I and II, oral cavity, testis, and intestines (6-8). Lung epithelial cells express high levels of ACE-2, which positively correlates with airway epithelial differentiation [59]. ACE-2 is involved in pulmonary hypertension (PH) and fibrosis [59]; and has been implicated in acute lung injury (ALI) by inducing an imbalance in RAS. Evidence reveals that in ALI, supplementation with ACE-2 or inhibition of Ang-II improves outcomes and that a decrease in pulmonary ACE-2 and increase in Ang-II levels aggravate viral-induced ALI [60]. In addition to its role in cardiovascular physiology, ACE-2 is a receptor for the coronavirus linked to SARS [61,62]. Surprisingly, involvement of ACE-2 in ARDS, which is triggered by multiple diseases including SARS-CoV and SARS-CoV-2 infections, has been established in multiple animal models [63]. ACE-2 KO mice exhibit severe pathology of ARDS and age-related loss of ACE-2 in the lungs correlates with increased mortality and worsened phenotype in elderly patients with COVID-19 [60,64]. Exogenous administration of ACE-2 in patients with ARDS did not show any difference in oxygenation index or clinical outcomes, although there was a trend of decreasing IL-6 concentrations [65]. A recent study showed that upregulation of *ZEB1* reduced ACE-2 expression, which in turn downregulated claudins, thus, increasing risk and pathogenesis of edema and ARDS in COVID-19 patients [66]. Collectively, these studies unequivocally establish the conceptual framework that ACE-2 is a central player in normal pulmonary function, and its imbalance leads to pulmonary diseases.

On the other hand, in-situ analyses in humans and mice, showed that at both protein and mRNA levels TMPRSS2 is expressed in the epithelia lining the salivary glands, gastrointestinal (stomach, colon, small intestine and pancreas), urogenital (kidney, prostate and ovary), and respiratory tracts including the bronchi and bronchioles, lung, but not in alveolar epithelium [67,68,69,70]. In prostate cells, TMPRSS2 is a constituent of the normal seminal fluid and present in the secretory epithelium [68]; moreover, its expression is controlled by androgens in prostate cancer cells and tissues [56]. In parallel, the expression of TMPRSS2 along with human airway trypsin-like protease (HAT) in the human lung is demonstrated to support spread of the human influenza virus [71,72]. Furthermore, TMPRSS2 and TMPRSS4 stimulate hemagglutinin of the highly pathogenic 1918 influenza virus [73], indicating the role of TTSPs in stimulating influenza virus in the human host. TMPRSS2 controls sodium currents in lung epithelial cells through proteolytic cleavage of the epithelial sodium channel [58]. However, TMPRSS2 expression is inactivated via homologous recombination due to serine protease domain disruption. Moreover, an in-vivo study in mice showed that TMPRSS2 deficiency did not affect growth or survival of murine embryonic development with no anomalies in organ histology and function [74]. Another study showed that TMPRSS2 depletion resulted in frailer, or delayed, inflammatory chemokine and cytokine responses mediated by Toll-like receptor 3 (TLR3) [28].

Proteases are vital components of respiratory host defense; in the healthy lung they are involved in regulating tissue homeostasis [75]. Along with serine proteases, cysteine proteases and matrix metalloproteinases (MMPs) are dominantly present in the lung; enhanced activity of proteases is linked with lung damage and the development of chronic lung diseases including emphysema and chronic obstructive pulmonary disease (COPD) [76,77]. TMPRSS2 cleavage activity plays a vital role in enhancing viral propagation of H7N9 influenza virus, H1N1 subtype influenza virus, SARS-CoV and MERS-CoV [27,78,79,80]. TMPRSS2 stimulates influenza virus by cleaving hemagglutinin, indicating that the enzyme can play a role in viral invasion of human airways [71,72]. Similarly, in the case of human metapneumovirus (HMPV) which is responsible for bronchiolitis and pneumonia [81], TMPRSS2 stimulates the HMPV F protein allowing fusion and cleavage, thus enhancing virus propagation [82]. In-vivo study using mice lacking TMPRSS2 displayed suppressed or delayed inflammatory chemokine and cytokine responses stimulated by TLR3 [83]. Activators of the cytokine response such as interleukin-6 (IL–6), tumor-necrosis factor- α (TNF-α) and interferon- γ (IFN-γ) are involved in the onset of ARDS and has been detected in severe COVID–19 patients [83].

**Figure 1:**
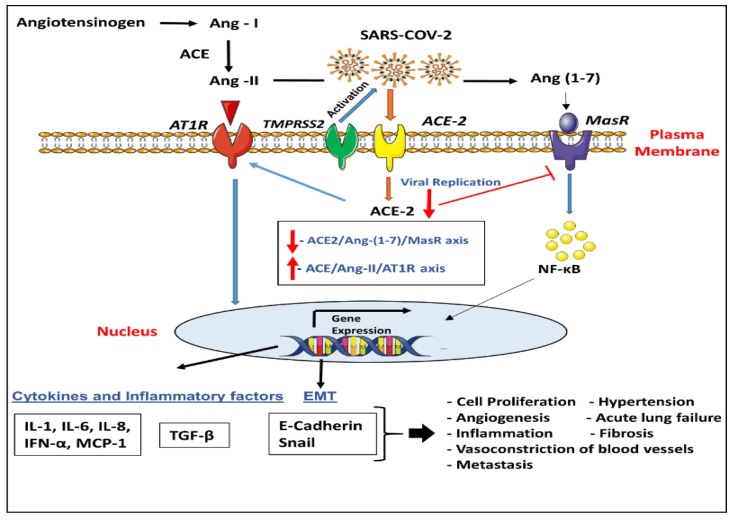
Schematic model for the effects of angiotensin converting enzyme II (ACE-2)/transmembrane serine protease 2 (TMPRSS2) axis in severe acute respiratory syndrome coronavirus 2 (SARS-CoV-2) action and tumor lung tissues. Activation of renin–angiotensin system cascade potentially leading to angiotensinogen enzymatic cleavage to Angiotensin I (Ang- I). Additionally, increase ACE abundance will potentially enhance production of the biologically active Ang-II that may lead to increase activation of Angiotensin type 1 receptor (AT1R) upon Ang-II binding. ACE-2 converts Ang-I to Ang (1-7) which acts through Mas receptor to protect from acute lung failure promoted through AT1R activation. However, it is still unknown how it may also enhance tumor development by stimulating vascularization. On the other hand, the “spike” attachment protein for the current SARS-CoV-2 coronavirus uses the cellular attachment factor, ACE-2 and requires TMPRSS2 cell protease for activation. This scenario antagonizes the protective role of ACE-2 and thus leads to severe lung injury and acute lung failure. Together, these upregulations have the potential to mediate intracellular signaling pathways and stimulate the production of different cytokines, inflammatory, and growth components such as the formation of transforming growth factor (TGF)-β and NF-κB to promote tumorigenesis and epithelial–mesenchymal transition (EMT) properties including; proliferation, angiogenesis, fibrosis, migration, and invasion.

In healthy individuals, when SARS-CoV-2 infects cells expressing ACE-2 and TMPRSS2, active replication releases the virus resulting in pyroptosis, an inflammatory form of programmed cell death [84]; the initial inflammatory response attracts virus-specific T cells to the site of infection, where the infected cells are destroyed prior to virus spread, thus causing insignificant lung damage. However, in immunocompromised patients, SARS-CoV-2-linked pyroptosis releases damage-associated patterns (ATP, DNA, and ASC oligomers) which are recognized by neighboring alveolar epithelial cells, endothelial cells and alveolar macrophages, stimulating release of pro-inflammatory cytokines and chemokines (IL-6, IP-10, IL-1β, macrophage inflammatory protein 1 (MIP1α and MIP1β), TNF, and MCP1) into the blood of SARS-CoV-2-infected patients [17,85,86,87]. These proteins attract monocytes, macrophages and T cells to the site of infection [88,89], thus, stimulating inflammation and accumulation of immune cells in the lungs, causing further production of pro-inflammatory cytokines thereby promoting lung damage [90] (Figure 1). Uncontrolled inflammatory cell infiltration increases lung injury caused by SARS-CoV-2, resulting in diffuse alveolar damage [88,89], thus, restricting gas exchange efficacy in lungs and affecting blood oxygenation levels; thereby leaving the lungs highly prone to secondary infections [90]. Additionally, along with local damage, enhanced levels of cytokines induce septic shock and multi-organ failure [91].

The primary site of SARS-CoV-2 infection is the human airway epithelium [28]. Infection by SARS-CoV results in loss of TMPRSS2 expression in the airways and induces acute lung injury [28]. An in-vivo study showed the spread of SARS-CoV in an infected mouse was stimulated by TMPRSS2 [92]. Furthermore, another in-vivo study involving TMPRSS2-deficient mice, showed lack of CoV replication in the lungs as well as bronchioles [28]. However, in contrast, CoV spread and inflammatory infiltration was observed in the alveoli in TMPRSS2-deficient mice; indicating the role of other serine proteases and CatL in triggering SARS-CoV and MERS-CoV to migrate to alveolar areas [28]. In a very recent study, engineered kidney cell line, VeroE6/TMPRSS2 showed significant proneness to SARS-CoV-2 infection, indicating a vital role of TMPRSS2 in SARS-CoV-2 [93]. During SARS-CoV-2, the insertion sequence (680-SPRR-683) enhances TMPRSS2 cleavage activity, thus, increasing the viral infectivity [94]. A recent study showed co-expression of TMPRSS2 and ACE-2 in the absorptive enterocytes, esophageal upper epithelial cells and lung AT2 cells, suggesting a significant role of TMPRSS2 in COVID-19 infection [94].

As the interaction between coronaviruses and the host cell is mediated by binding of the viral spike (S) protein to specific cell receptors of the host. This step is a major determinant of the viral host and a tissue tropism. Structural studies were performed on different species revealing some mechanisms of the SARS-CoV-2 use of cross-species receptor. The results indicate possible interspecies transmission of SARS-CoV-2 and suggest additional surveillance in other animal populations [95].

In this regard, TMPRSS2 plays a vital role during viral entry, as the S protein of SARS-CoV uses the endosomal cysteine proteases cathepsin B and L (CatB/L) for S protein priming in TMPRSS2 receptor [96]; however, priming of S protein by TMPRSS2 but not CatB/L is crucial for viral entry into target cells and viral spread in the infected host [28,92]. Furthermore, TMPRSS2 cleaves SARS-S at residue R667, which is significantly linked with the activation of SARS-S for cell to cell fusion [97]; R667 is frequently present in several cleavage motifs and is cleaved during S protein biogenesis [23]. Another residue, R797 is also vital for S protein activation by TMPRSS2 and is often cleaved during viral entry [23]. A recent study indicated that the extra additional arginine residues linked structure projected from the protein surface enhances the recognition and cleavage activity of TMPRSS2, thus, increasing the viral infectivity of SARS-CoV-2 [94]. However, a few studies indicate presence of four inserts in the S protein of SARS-CoV-2 due to artificial modification [94].

In a study by Lukassen et al. [98] RNA sequencing was used to analyze the expression of both ACE-2 and TMPRSS2 in lung tissue and single cells obtained from normal subsegmental bronchial branches and lung tissues that were resected from 16 patients with lung cancer (6 males and 10 females; mean age ~50 years) [98]. The study reported a significant expression of ACE-2 in subsegmental bronchial branches (in transient secretory cells) while TMPRSS2 was expressed in both lung tissues and bronchial branches [98]. Intriguingly, these bronchial transient secretory cells were involved in pathways related to RHO GTPase function and viral processes, indicating increased susceptibility for SARS-CoV-2 infection. Although ACE-2 expression was not sex or age-dependent at a single cell level, the authors found a trend for age dependency when all lung cell types are summed up from female patients. The authors concluded that further larger studies involving both healthy and infected patients of both sexes are required for a better understanding of vulnerability of different age groups to SARS-CoV-2 [98]. Another study implicated the co-expression of TMPRSS2 and ACE-2 in the esophageal upper epithelial cells and lung AT2 cells, in COVID-19 infection [94]. A recent investigation used the LungMAP website, GEPIA2 software and the lung cancer explorer (database consisting of 6700 patients) to analyze ACE-2 and TMPRSS2 expression in lung development and lung cancer, respectively [99]. However, the majority of those with upregulated ACE-2 expression were more prone to developing COVID-19 disease [99].

A study by Pinto et al. [100] analyzed the expression of ACE-2 in more than 700 lung transcriptome samples of patients with diseases including hypertension, diabetes, lung diseases (COPD or pulmonary arterial hypertension) as well as cancer; as compared to healthy individuals, ACE-2 was upregulated in patients with comorbidities [100,101], indicating patients presenting with these disease are at a higher risk of developing COVID-19. Furthermore, ACE-2 expression was higher in cancer as compared to normal lung tissue [100]. In this study, network analyses identified several candidate regulators of ACE-2 in the human lung; the majority of these genes were associated with histone modifications as well as epigenetic regulation of gene transcription (HAT1, HDAC2, and KDM5B) [100]. HAT and HDAC are involved in chromatin modification and DNA condensation, thus, allowing gene transcription and upregulation of ACE-2 expression [100], indicating their role in lung disease including COPD and lung cancer. Other genes included ADAM10 and TLR3; ADAM10 was found to regulate ACE2 cleavage in the human airway epithelia [102], while, TLR3 was involved in the innate response to SARS-CoV or MERS-CoV infection [103].

## 4. Key Entry Genes of SARS-CoV-2 and Human Cancers Including Lung Cancer

A recent study analyzed the risk for developing COVID-19 in patients with cancer; they concluded that these patients were at a higher risk of developing COVID-19 with poorer outcomes than individuals without cancer [104]. Research into the link between the RAS and cancer has accelerated greatly over the past years due to the growing evidence of local RAS signaling being deregulated in pathological tissues and the prominent role for the angiotensin receptors in tissue remodeling. As a result of the effects of ACE and the RAS as a whole, numerous studies have shown the association between ACE function and metabolic diseases such as diabetes as well as various cancers such as pancreatic and breast cancer [105,106,107]. Thus, drugs used to target RAS might also prove effective against cancer.

There is a large body of evidence supporting the role of ACE-2 axis in disease and cancer [108,109,110,111]. The ACE-2 enzyme serves to counterbalance the effects of the ACE within the RAS [112]. Recent research has begun to address whether the stimulation of ACE-2 function, or overexpression of ACE-2 in diseased states is linked to positive effects [113]. Activation of the ACE-2 axis using Ang (1-7) in cancer studies showed promising anticancer effects with disruption of growth-promoting signals as well as decreased angiogenesis, inflammation and metastasis of breast, prostate and hepatocellular carcinoma cells [114,115]. ACE-2/Ang (1-7) axis overexpression has also been reported to inhibit cell proliferation in pancreatic cancer cell lines, BxPC3 and SW1990, reduce epithelial–mesenchymal transition (EMT) and inhibits the migration and invasion of non-small cell lung cancer (NSCLC) cells, A549 [116,117,118]. A recent study reported that downregulation of the ACE-2 axis promotes breast cancer cell metastasis via increased calcium signaling [119]. In addition, the anticancer activity of Ang (1-7) has also been described using in-vitro and in-vivo model systems [120,121,122]. Interestingly, it is suggested that upon SARS-CoV binding to ACE-2 followed by cell entry and replication, ACE-2 is downregulated [123], causing an imbalance between ACE/Ang-II/AT1R axis and ACE-2/Ang (1-7)/MasR axis. These events can promote severe lung injury and acute lung failure as well as enhance cancer pathogenicity through several pathways [124,125]. In this regard, SARS-CoV-2-induced cell infection, promote molecular changes, characterized by enhanced expression of *ZEB1* and *AXL*, with comparatively reduced miR-200 levels and glutamine synthesis, indicating epithelial–mesenchymal transition, a key feature of cancer onset and progression [66].

In human prostate and colon cancers, TMPRSS2 protein was found to be located on the apical membrane of secretory epithelia as well as in the lumen of the glands [56]. In prostate cancer, a protease domain of around 32-kDa has been identified, indicating TMPRSS2 to be partly activated [56]. Similarly, TMPRSS2 mRNA expression is upregulated in androgen-activated prostate cancer cells [67]; elevated TMPRSS2 mRNA expression is suggested to be facilitated by the androgen receptor [56]. Furthermore, both in-vitro and in-vivo studies demonstrate treatment by androgen enhanced TMPRSS2 zymogen activation, thus implicating the role of TMPRSS2 in the onset and progression of prostate cancer in an androgen-dependent manner [126]. Gene fusion between *TMPRSS2* and members of the E26 transformation specific (ETS) transcription factor family (*ERG* or *ETV*) is another mechanism by which TMPRSS2 plays a role in prostate cancer progression [127]. During gene fusion, the 5′-untranslated region of TMPRSS2 is merged with the transcription factors, *ERG* or *ETV* genes which enhance prostate cancer progression and invasion [128]. The most frequent gene fusion in prostate cancer is the TMPRSS2–ERG gene fusion, which comprise around 50% of prostate cancers [128]. Molecular research demonstrated dysregulated ERG expression to disrupt normal androgen receptor signaling and trigger epigenetic pathways, thus promoting tumorigenesis [128]. The G-protein-coupled PAR-2 (protease-activated receptor-2) is expressed in prostate cancer cells [129,130,131]. TMPRSS2 stimulates PAR-2 leading to upregulated levels of MMPs-2 and -9, thus, promoting inflammation, invasion, and metastases [132,133].

TMPRSS2 is also expressed in colon cancer, hepatocellular carcinoma [134], human nasal and tracheal mucosa, distal airways, and lung [58,67,71,135]. Other TTPs are also involved in cancer; while, TMPRSS4 is expressed in lung cancer tissue [136] and the human trachea [135], TMPRSS11D is expressed in human bronchi and tracheae [135,137,138]. As compared to small-cell prostate carcinoma, the TMPRSS2-ERG gene fusion is frequently absent in any small cell carcinoma of the urinary bladder or lung; this can aid in differentiating small cell carcinoma of prostatic origin from non-prostatic origins [139].

More specifically, vis-à-vis lung cancer and COVID-19, it is well-established that proteolytic breakdown of the ECM components (collagens, laminins and elastin) result in severe lung damage (164). Cancer patients are more likely to develop COVID-19 than the general population due to malignancies-related chronic immunosuppressive and anti-cancer therapy [85,104]. Patients with lung cancer are more susceptible to COVID–19 since they are typically smokers and old patients with eventual occurrence of treatment-related immune deficiency [140,141,142]. Smokers increase their risk of lung disease, including lung cancer, and are likely to be more vulnerable to COVID-19. It has been shown that the histological changes and inflammation in ARDS resulted from smoking-induced lung injury were presumably due to an irregular activation of ACE/ACE-2 pathway imbalance [63]. In order to study the role of ACE-2 in lung injury, a mouse model of induced lung injury by cigarette smoke exposure for 1 to 3 weeks revealed that ACE-2 deficiency influences STAT3 phosphorylation and MMPs activation to promote more pulmonary inflammation in the development of lung injury [143].

The ERK/MAPK/JNK signaling pathways are involved in regulating several processes including cellular growth, proliferation, differentiation and apoptosis; which are important processes vital for the onset and progression of tumors, including lung cancer [144,145,146,147].

Additionally, several studies reported phosphorylation of p38/ERK/JNK in SARS-CoV-infected Vero E6 cells [148,149,150]; while, activated p38 has been indicated to play a role in lung cancer development [151]. Also, using SARS-CoV-2-transfected lung cancer cell line, A549, it was pointed out that SARS-CoV S protein, or SARS-CoV virus-like particles, can activate ERK [152]. Similarly, cells transfected with MERS-CoV and HCoV-229 also trigger the ERK pathway [153]. Moreover, upregulated expression of SARS-CoV S protein in kidney cell line, 293T, can phosphorylate JNK via protein kinase C epsilon in a calcium-independent pathway [154]. While, apoptosis induced by SARS-CoV N protein upregulation is JNK dependent [153], JNK activation also stimulates IBV-induced apoptosis [155,156], indicating, a pro-apoptotic role of JNK during early SARS-CoV infection and a pro-survival role in continual SARS-CoV-infected cells.

The clinical aspects of cancer patients with COVID-19 have remained relatively unknown until now. Considering the related clinical symptoms of lung cancer such as fever, cough, and dyspnea with SARS-CoV-2 infection, an effective COVID-19 screening program may enable early diagnosis and proper treatment in order to significantly reduce the risk of disease and mortality [157]. A study of 18 cancer patients from a nationwide 2007 COVID-19 cohort showed that cancer patients were at greater risk of serious adverse incidents than non-cancer patients [104]. Another study with 28 cancer patients showed that lung cancer patients developed severe baseline lung function, endurance, and anoxia more rapidly with COVID-19 [85]. Based on these relevant findings, appropriate management of lung cancer patients remain unclear, and a better understanding is needed to reduce the number of cancer-infectious related targets throughout the COVID-19 era. Although it seems logical to postpone or delay the delivery of cancer treatment in certain cases, the eventual outcomes, risk, or benefits of those changes are still to be assessed.

A very recent study by Kong (2020), analyzed the differential expression of ACE-2 and TMPRSS2 in two common types of lung cancers, lung adenocarcinoma (LUAD) and lung squamous cell carcinoma (LUSC), and their correlation with prognosis and SARS-CoV-2 infection [99]. In this study, gene expression of TMPRSS2 was reduced in LUSC as compared to LUAD; however, expression of ACE-2 was enhanced in LUAD but not in LUSC [99]. Furthermore, in comparison to ACE-2 expression in normal lung tissue, TMPRSS2 expression was enhanced; this differential expression was more profound in LUAD and significantly correlated with pathological stages and subtypes [99]. The study also suggested that lung cancer patients, especially LUAD, were more prone to develop COVID-19 disease [99]. Data using the lung cancer cell line, Calu-3 cells transfected with SARS-CoV as well as lung cancer samples showed less elevated TMPRSS2 expression than ACE-2 expression, which was highly expressed [158].

The direct implications of ACE-2 inhibition in COVID-19 patients with lung cancer remain elusive, and clinical evidence is desperately needed to determine the relative benefits and risks associated with usage of these medications [60]. Nonetheless, modulation of ACE-2 in patients already infected by SARS-CoV-2 may be an effective therapeutic option in addressing the viral-mediated RAS imbalance and is currently under investigation in several clinical trials.

## 5. Emerging Therapeutic Approaches for ACE-2

Pharmacological RAS blockade agents, angiotensin receptor blockers (ARBs), in particular, are capable of modulating both systemic and tissue RAS, simultaneously increasing ACE-2 expression and activity in experimental models. Generally, mechanisms behind the augmentation of *ACE-2* mRNA levels by ACE inhibitors and ARBs require further characterization. ARBs enhance the expression/activity of Ang (1-7) that is tissue-defensive and prevents Ang-II-induced acute lung injury and inflammation [159,160]. To date, however, no compelling scientific data have been found linking ACE inhibitors and/or ARBs with the incidence and mortality of COVID-19 [161].

**Figure 2:**
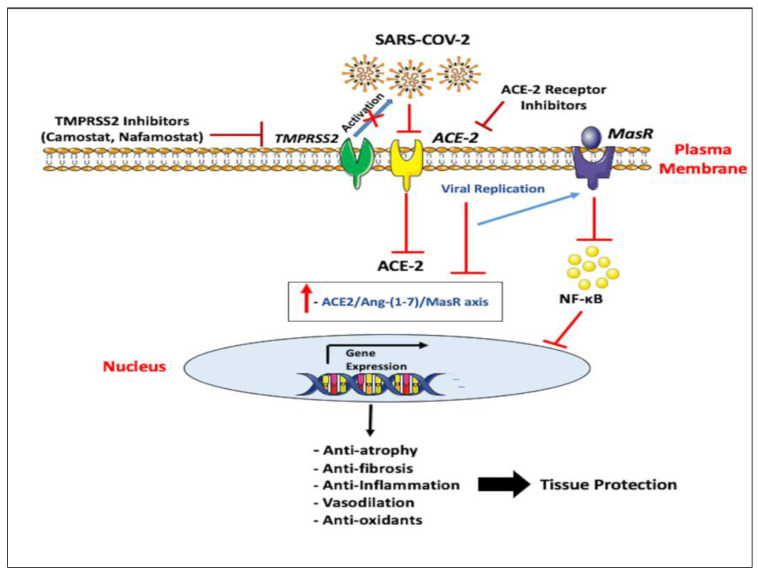
Potential therapeutic approaches to address ACE-2-mediated COVID-19 following SARS-CoV-2 infection. ACE-2 converts Ang-I to Ang (1-7) which acts through Mas receptor to protect from acute lung failure. On the other hand, the “spike” attachment protein for the current SARS-CoV-2 coronavirus uses the cellular attachment factor, (ACE-2) and requires TMPRSS2 cell protease for activation. Therapeutic approaches include TMPRSS2 inhibitors (Camostat and Nafamostat) that inhibit priming of the spike protein as well as blocking the surface ACE-2 receptor using anti-ACE-2 antibody or peptides which decrease viral entry into cells through competitively binding with SARS-CoV-2 thereby reducing viral spread and protecting the lungs from injury through its unique enzymatic function.

While, AT1R inactivation with antagonists has successfully reduced tumor growth of cancers including gastric, colorectal, prostate, renal, breast, and NSCLCs [162,163,164,165]. Current pharmacotherapies aim to achieve multilevel RAS inhibition through distinct modes of action. Although ACE-2 is not the direct cellular target of these therapies, Ace-2 gene transcription, translation, and ultimately catalytic activities are modified due to the intricate nature of the RAS [60]. Blocking the ACE/Ang-II/AT1R axis through modulating the actions of Ang-II potentiates the effects of ACE-2 as the endogenous RAS counter-regulator based on the fact that Ang-II can regulate ACE-2 expression through AT1R [166]. The SARS and SARS-CoV-2 coincidences using the ACE-2 receptor allow the possibility of using and applying the detailed investigations of coronavirus entry to treat COVID-19. According to literature on SARS, various possible ACE-2 blocking techniques have proven successful in avoiding infection in SARS models which can be effective against COVID-19 (Figure 2).

On the other hand, one approach is to provide an ACE-2 binding agent to patients where the host ACE-2 protein is not altered thereby diminishing the risk associated with this therapeutic agent. Two recognized solutions are available for ACE-2 binding agents. The first one is utilizing the tiny SARS-S receptor-binding domain (RBD), the main domain that attaches to the ACE-2 receptor during entry [167]. Conversely, the RBD protein may be added to the expanded circulation fragment of Fc, which was developed as an analogous of 212 amino acid domains from MERS. It is known that if the RBD-Fc fusion attaches to human cells, the cytotoxicity of Fc domain will be eliminated by introducing mutations to disable Fc receptor binding [168]. A second approach is to supply an antibody that binds to the ACE-2 receptor to avoid infection with SARS-CoV-2. This approach has shown that the viral entry and replication are effectively blocked [169]. Although no ACE-2 sequence of antibodies is reported, monoclonal and associated hybridoma sequences can be coned. This approach is better than S-protein neutralization as no possible viral escape from ACE binding antibodies could be detected [170]. A single chain-variable-fragment (scFv) that binds to ACE-2 can be used through neutralization to increase the concentration of anti-ACE-2 upon local administration in infected lungs. Administration of SARS RBD-Fc fusion protein in murine lung tissues exacerbates the alveolar after ACE-2 interactions, which usually mitigate acute pulmonary injury [64]. This indicates the importance of using ACE-2 binding strategy early-on during infection or as a prophylaxis to prevent the initial viral infection. Collectively, these possible complications will need to be examined in clinical research.

While, it is clear that a far more effective approach would be to build an antibody-like agent that would attach to the coronavirus itself rather than protect the cells from infection. With this approach, it is recommended to use a soluble form of the ACE-2 receptor that will bind to the SARS-CoV-2 protein to neutralize the virus. Soluble ACE-2 receptor has been shown to prevent SARS from infecting cells in culture [169]. In order to use ACE-2 to treat COVID-19 patients, it is preferable to treat patients with soluble ACE-2 fused to Fc immunoglobulin domain (ACE-2-Fc). Previous studies have shown that fusion of human IgG1 to an ACE-2 extracellular domain was successful in neutralizing SARS-coronavirus in-vitro [171]. This study also offers proof that ACE-2-Fc could likewise inhibit SARS-CoV-2 in-vitro and possibly in patients.

Finally, it is evident that more focus has been given to ACE-2, only a few studies have considered TMPRSS2; suggesting that more research need to focus on TMPRSS2 which has a vital role along with ACE-2 in SARS-CoV-2 infection and can pave the way for therapeutic intervention in COVID–19.

## 6. Emerging Therapeutic Approaches for TMPRSS2

Of the various factors regulating SARS-CoV-2 entry, TMPRSS2 is the most favorable candidate for transcriptional inhibition as TMPRSS2 is required for SARS-CoV-2 entry to host cells and its expression levels are linked with lung disease severity.

In this context, blocking TMPRSS2 enables targeting influenza virus along with coronavirus, in addition to other respiratory viruses (Figure 2). TMPRSS2 enhances fusion proteins of some paramyxoviruses such as metapneumovirus [82], trypsin-dependent parainfluenza subtypes and Sendai virus [172]. Due to the acute role of TMPRSS2 as a host cell factor for viral infections [27,28,92,158], serine protease inhibitors (Camostat, Nafamostat, and Leupeptin) have been implicated in the antiviral therapeutic strategy targeting TMPRSS2 with high antiviral activities [92,173,174]. Along with camostat, aprotinin also blocks replication of the influenza virus in the human airway epithelial cells and releases cytokines (IL-6 and TNF-α) into cell supernatants [135]. The serine protease inhibitor, Camostat mesylate, commonly known as Foypan, is an approved drug in Japan for use in pancreatic inflammation; it is found to inhibit TMPRSS2 activity [92,158], suggesting use of the compound or related ones with possibly enhanced antiviral activity [174] might be considered as a therapeutic target for SARS-CoV-2-infected patients (Figure 2). Furthermore, in-vivo data showed efficiency of camostat in preventing mice from developing lethal infection by SARS coronavirus [92].

Nafamostat mesylate, also known as Fusan, is another serine protease inhibitor that acts as anti-coagulant with few anti-cancer and anti-viral properties [174]. Very recently, Nafamostat was found to inhibit fusion of the viral envelope with host cell surface membranes, the initial step in SARS-CoV-2 infection [175], thus, suggesting efficacy in inhibiting SARS-CoV-2 virus entry and spread (Figure 2). In comparison to Camostat, Nafamostat blocked SARS-CoV-2 S protein-initiated fusion at a concentration less than one-tenth as needed for Camostat, indicating Nafamostat as the most effective drug against SARS-CoV-2 S protein-initiated fusion in clinical practice [175]. Since, Nafamostat is administered intravenously; it is assumed that blood concentration of Nafamostat post-administration would exceed the experimental concentration to block SARS-CoV-2 S protein-initiated fusion [175]. In contrast, Camostat is orally administered and hence, it is indicated that blood levels may be inferior to Nafamostat, suggesting Nafamostat to have a higher preventive role in SARS-CoV-2 upon entering human cells [175].

Also, earlier studies analyzed the synergistic effect of airway protease inhibitors with conventional antiviral drugs to reduce the risk of resistance. Synergy of serine protease inhibitor, BAPA (benzylsulfonyl-d-Arg-Pro-4-amidinobenzylamide) with oseltamivir significantly inhibited replication of influenza virus in human airway epithelial cells at lower concentrations in comparison to treatment with each inhibitor alone [176].

Several FDA approved drugs are involved in blocking host proteins which regulate entry of SARS-CoV-2 into host cells. Of these, the estrogen-related compounds (estradiol and genistein) and the androgen receptor antagonist (enzalutamide) block TMPRSS2 [177,178,179], which is required for SARS-CoV-2 spike protein priming and appear to be the most promising repurposing candidates for symptom amelioration in COVID-19 patients. Commonly approved drugs which are involved in the same pathway can also be plausible candidates to transcriptionally suppress TMPRSS2, including dutasteride and finasteride [180,181]. Furthermore, in-vivo study using female mice-infected with SARS-CoV showed that treatment involving ovariectomy or estrogen receptor antagonists, enhanced TMPRSS2 expression resulting in lethality [182]. Finally, and since there is a lack of antiviral medication for these paramyxoviruses and coronaviruses, broader-acting airway protease inhibitors are needed to pave the way for therapeutic intervention.

## 7. Conclusions

As of 7 July 2020, there are more than 11.5 million infected people with SARS-CoV-2 virus, that caused around half a million deaths with an excess of 6.6 million reported recoveries [183]. Meanwhile, it is evident that numerous questions regarding the coronavirus infection and pathogenesis remain unanswered. Indeed, patients with cancer, particularly lung cancer, are at a higher risk of developing severe complications following their infection by SARS-CoV-2, hence important measures have to be considered to minimize infection risk for this group of patients. Moreover, extensive studies on molecular and cellular levels are needed to understand the mechanism of action of this virus which may allow the development of new therapies to manage the infection of SARS-CoV-2; specially since there is no vaccine available to prevent this viral infection. In this context, structural experiments of SARS-CoV-2 S-Proteins binding to ACE-2 and TMPRSS2, the key entry genes in infected cells, should contribute to better understanding of the structural/functional correlations of this novel coronavirus that would enable targeted therapies via blocking or manipulating one or both of them. On the other hand, developing in-vitro and in-vivo models including conditional double transgenic/knockout of ACE-2 and TMPRSS2 could be one of best models to understand to role of these genes in the infection process which also can help to develop new therapies based on the shutdown of one or both of these genes.

## References

[1] Ferlay J., Colombet M., Soerjomataram I., Mathers C., Parkin D.M., Piñeros M., Znaor A., Bray F. (2019). Estimating the global cancer incidence and mortality in 2018: GLOBOCAN sources and methods. Int. J. Cancer.

[2] Lemjabbar-Alaoui H., Hassan O.U., Yang Y.-W., Buchanan P. (2015). Lung cancer: Biology and treatment options. Biochim. Biophys. Acta.

[3] Youlden D.R., Cramb S.M., Baade P.D. (2008). The International Epidemiology of Lung Cancer: Geographical distribution and secular trends. J. Thorac. Oncol..

[4] Sasco A.J., Secretan M.B., Straif K. (2004). Tobacco smoking and cancer: A brief review of recent epidemiological evidence. Lung Cancer.

[5] Littman A.J., Jackson L.A., Vaughan T.L. (2005). Chlamydia pneumoniae and lung cancer: Epidemiologic evidence. Cancer Epidemiol. Biomark. Prev..

[6] Lanoix J.-P., Pluquet E., Lescure F.X., Bentayeb H., Lecuyer E., Boutemy M., Dumont P., Jounieaux V., Schmit J.L., Dayen C. (2011). Bacterial infection profiles in lung cancer patients with febrile neutropenia. BMC Infect. Dis..

[7] Greathouse K.L., White J.R., Vargas A.J., Bliskovsky V.V., Beck J.A., von Muhlinen N., Polley E.C., Bowman E.D., Khan M.A., Robles A.I. (2018). Interaction between the microbiome and TP53 in human lung cancer. Genome Biol..

[8] Robinson L.A., Jaing C.J., Pierce Campbell C., Magliocco A., Xiong Y., Magliocco G., Thissen J.B., Antonia S. (2016). Molecular evidence of viral DNA in non-small cell lung cancer and non-neoplastic lung. Br. J. Cancer.

[9] Cardona A., Ruiz-Patiño A., Ricaurte L., Rojas L., Zatarain-Barrón Z.L., Arrieta O., Rosell R. (2018). Human Papillomavirus Infection and Lung Cancer. Current Perspectives in Human Papillomavirus.

[10] Syrjänen K.J. (2002). HPV infections and lung cancer. J. Clin. Pathol..

[11] Kheir F., Zhao M., Strong M.J., Yu Y., Nanbo A., Flemington E.K., Morris G.F., Reiss K., Li L., Lin Z. (2019). Detection of Epstein-Barr Virus Infection in Non-Small Cell Lung Cancer. Cancers.

[12] Zur Hausen H. (2009). The search for infectious causes of human cancers: Where and why. Virology.

[13] Zhang X., Zhang Z., Zheng B., He Z., Winberg G., Ernberg I. (2013). An update on viral association of human cancers. Arch. Virol..

[14] Vijayanand P., Wilkins E., Woodhead M. (2004). Severe acute respiratory syndrome (SARS): A review. Clin. Med..

[15] Ramadan N., Shaib H. (2019). Middle East respiratory syndrome coronavirus (MERS-CoV): A review. Germs.

[16] Kumar D., Malviya R., Kumar Sharma P. (2020). Corona Virus: A Review of COVID-19. EJMO.

[17] Huang C., Wang Y., Li X., Ren L., Zhao J., Hu Y., Zhang L., Fan G., Xu J., Gu X. (2020). Clinical features of patients infected with 2019 novel coronavirus in Wuhan, China. Lancet.

[18] Chan J.F.-W., Yuan S., Kok K.-H., To K.K.-W., Chu H., Yang J., Xing F., Liu J., Yip C.C.-Y., Poon R.W.-S. (2020). A familial cluster of pneumonia associated with the 2019 novel coronavirus indicating person-to-person transmission: A study of a family cluster. Lancet.

[19] Zhou P., Yang X.-L., Wang X.-G., Hu B., Zhang L., Zhang W., Si H.-R., Zhu Y., Li B., Huang C.-L. (2020). A pneumonia outbreak associated with a new coronavirus of probable bat origin. Nature.

[20] Zhu N., Zhang D., Wang W., Li X., Yang B., Song J., Zhao X., Huang B., Shi W., Lu R. (2020). A Novel Coronavirus from Patients with Pneumonia in China, 2019. N. Engl. J. Med..

[21] Zhang T., Wu Q., Zhang Z. (2020). Probable Pangolin Origin of SARS-CoV-2 Associated with the COVID-19 Outbreak. Curr. Biol..

[22] Shen L.W., Mao H.J., Wu Y.L., Tanaka Y., Zhang W. (2017). TMPRSS2: A potential target for treatment of influenza virus and coronavirus infections. Biochimie.

[23] Millet J.K., Whittaker G.R. (2015). Host cell proteases: Critical determinants of coronavirus tropism and pathogenesis. Virus Res..

[24] Reguera J., Mudgal G., Santiago C., Casasnovas J.M. (2014). A structural view of coronavirus–receptor interactions. Virus Res..

[25] Belouzard S., Millet J.K., Licitra B.N., Whittaker G.R. (2012). Mechanisms of coronavirus cell entry mediated by the viral spike protein. Viruses.

[26] Riordan J.F. (2003). Angiotensin-I-converting enzyme and its relatives. Genome Biol..

[27] Matsuyama S., Nagata N., Shirato K., Kawase M., Takeda M., Taguchi F. (2020). Efficient Activation of the Severe Acute Respiratory Syndrome Coronavirus Spike Protein by the Transmembrane Protease TMPRSS2. J. Virol..

[28] Iwata-Yoshikawa N., Okamura T., Shimizu Y., Hasegawa H., Takeda M., Nagata N. (2019). TMPRSS2 Contributes to Virus Spread and Immunopathology in the Airways of Murine Models after Coronavirus Infection. J. Virol..

[29] Heurich A., Hofmann-Winkler H., Gierer S., Liepold T., Jahn O., Pöhlmann S. (2014). TMPRSS2 and ADAM17 cleave ACE2 differentially and only proteolysis by TMPRSS2 augments entry driven by the severe acute respiratory syndrome coronavirus spike protein. J. Virol..

[30] Coutard B., Valle C., de Lamballerie X., Canard B., Seidah N.G., Decroly E. (2020). The spike glycoprotein of the new coronavirus 2019-nCoV contains a furin-like cleavage site absent in CoV of the same clade. Antivir. Res..

[31] Burkard C., Verheije M.H., Wicht O., van Kasteren S.I., van Kuppeveld F.J., Haagmans B.L., Pelkmans L., Rottier P.J., Bosch B.J., de Haan C.A. (2014). Coronavirus cell entry occurs through the endo-/lysosomal pathway in a proteolysis-dependent manner. PLoS Pathog..

[32] Millet J.K., Whittaker G.R. (2014). Host cell entry of Middle East respiratory syndrome coronavirus after two-step, furin-mediated activation of the spike protein. Proc. Natl. Acad. Sci. USA.

[33] Tigerstedt R., Bergman P. (1898). Niere und Kreislauf 1. Skand. Arch. Für Physiol..

[34] N Campbell K., Raij L., Mundel P. (2011). Role of angiotensin II in the development of nephropathy and podocytopathy of diabetes. Curr. Diabetes Rev..

[35] Peach M.J. (1977). Renin-angiotensin system: Biochemistry and mechanisms of action. Physiol. Rev..

[36] Dasgupta C., Zhang L. (2011). Angiotensin II receptors and drug discovery in cardiovascular disease. Drug Discov. Today.

[37] Ager E.I., Neo J., Christophi C. (2008). The renin–angiotensin system and malignancy. Carcinogenesis.

[38] Gul R., Ramdas M., Mandavia C.H., Sowers J.R., Pulakat L. (2012). RAS-mediated adaptive mechanisms in cardiovascular tissues: Confounding factors of RAS blockade therapy and alternative approaches. Cardiorenal Med..

[39] Donoghue M., Hsieh F., Baronas E., Godbout K., Gosselin M., Stagliano N., Donovan M., Woolf B., Robison K., Jeyaseelan R. (2000). A novel angiotensin-converting enzyme–related carboxypeptidase (ACE2) converts angiotensin I to angiotensin 1–9. Circ. Res..

[40] Tipnis S.R., Hooper N.M., Hyde R., Karran E., Christie G., Turner A.J. (2000). A human homolog of angiotensin-converting enzyme cloning and functional expression as a captopril-insensitive carboxypeptidase. J. Biol. Chem..

[41] Oudit G.Y., Penninger J.M. (2011). Recombinant human angiotensin-converting enzyme 2 as a new renin-angiotensin system peptidase for heart failure therapy. Curr. Heart Fail. Rep..

[42] Skeggs L.T., Kahn J.R., Shumway N.P. (1956). The preparation and function of the hypertensin-converting enzyme. J. Exp. Med..

[43] Garg M., Angus P.W., Burrell L.M., Herath C., Gibson P.R., Lubel J.S. (2012). The pathophysiological roles of the renin–angiotensin system in the gastrointestinal tract. Aliment. Pharmacol. Ther..

[44] Jiang F., Yang J., Zhang Y., Dong M., Wang S., Zhang Q., Liu F.F., Zhang K., Zhang C. (2014). Angiotensin-converting enzyme 2 and angiotensin 1–7: Novel therapeutic targets. Nat. Rev. Cardiol..

[45] Vickers C., Hales P., Kaushik V., Dick L., Gavin J., Tang J., Godbout K., Parsons T., Baronas E., Hsieh F. (2002). Hydrolysis of biological peptides by human angiotensin-converting enzyme-related carboxypeptidase. J. Biol. Chem..

[46] Abuohashish H.M., Ahmed M.M., Sabry D., Khattab M.M., Al-Rejaie S.S. (2017). Angiotensin (1-7) ameliorates the structural and biochemical alterations of ovariectomy-induced osteoporosis in rats via activation of ACE-2/Mas receptor axis. Sci. Rep..

[47] Santos R.A., e Silva A.C.S., Maric C., Silva D.M., Machado R.P., de Buhr I., Heringer-Walther S., Pinheiro S.V.B., Lopes M.T., Bader M. (2003). Angiotensin-(1–7) is an endogenous ligand for the G protein-coupled receptor Mas. Proc. Natl. Acad. Sci. USA.

[48] Iusuf D., Henning R.H., van Gilst W.H., Roks A.J. (2008). Angiotensin-(1–7): Pharmacological properties and pharmacotherapeutic perspectives. Eur. J. Pharmacol..

[49] Allred A.J., Diz D.I., Ferrario C.M., Chappell M.C. (2000). Pathways for angiotensin-(1—7) metabolism in pulmonary and renal tissues. Am. J. Physiol. Ren. Physiol..

[50] Hooper J.D., Clements J.A., Quigley J.P., Antalis T.M. (2001). Type II transmembrane serine proteases. Insights into an emerging class of cell surface proteolytic enzymes. J. Biol. Chem..

[51] Bugge T.H., Antalis T.M., Wu Q. (2009). Type II Transmembrane Serine Proteases. J. Biol. Chem..

[52] Appel L.F., Prout M., Abu-Shumays R., Hammonds A., Garbe J.C., Fristrom D., Fristrom J. (1993). The Drosophila Stubble-stubbloid gene encodes an apparent transmembrane serine protease required for epithelial morphogenesis. Proc. Natl. Acad. Sci. USA.

[53] Irving P., Troxler L., Heuer T.S., Belvin M., Kopczynski C., Reichhart J.-M., Hoffmann J.A., Hetru C. (2001). A genome-wide analysis of immune responses in Drosophila. Proc. Natl. Acad. Sci. USA.

[54] Paoloni-Giacobino A., Chen H., Peitsch M.C., Rossier C., Antonarakis S.E. (1997). Cloning of the TMPRSS2 Gene, Which Encodes a Novel Serine Protease with Transmembrane, LDLRA, and SRCR Domains and Maps to 21q22.3. Genomics.

[55] Jacquinet E., Rao N.V., Rao G.V., Zhengming W., Albertine K.H., Hoidal J.R. (2001). Cloning and characterization of the cDNA and gene for human epitheliasin. Eur. J. Biochem..

[56] Afar D.E., Vivanco I., Hubert R.S., Kuo J., Chen E., Saffran D.C., Raitano A.B., Jakobovits A. (2001). Catalytic cleavage of the androgen-regulated TMPRSS2 protease results in its secretion by prostate and prostate cancer epithelia. Cancer Res..

[57] Chen Y.-W., Lee M.-S., Lucht A., Chou F.-P., Huang W., Havighurst T.C., Kim K., Wang J.-K., Antalis T.M., Johnson M.D. (2010). TMPRSS2, a Serine Protease Expressed in the Prostate on the Apical Surface of Luminal Epithelial Cells and Released into Semen in Prostasomes, Is Misregulated in Prostate Cancer Cells. Am. J. Pathol..

[58] Donaldson S.H., Hirsh A., Li D.C., Holloway G., Chao J., Boucher R.C., Gabriel S.E. (2002). Regulation of the epithelial sodium channel by serine proteases in human airways. J. Biol. Chem..

[59] Rey-Parra G., Vadivel A., Coltan L., Hall A., Eaton F., Schuster M., Loibner H., Penninger J., Kassiri Z., Oudit G. (2012). Angiotensin converting enzyme 2 abrogates bleomycin-induced lung injury. J. Mol. Med..

[60] Gheblawi M., Wang K., Viveiros A., Nguyen Q., Zhong J.-C., Turner A.J., Raizada M.K., Grant M.B., Oudit G.Y. (2020). Angiotensin Converting Enzyme 2: SARS-CoV-2 Receptor and Regulator of the Renin-Angiotensin System. Circ. Res..

[61] Zheng Y.-Y., Ma Y.-T., Zhang J.-Y., Xie X. (2020). COVID-19 and the cardiovascular system. Nat. Rev. Cardiol..

[62] Barnard D.L., Kumaki Y. (2011). Recent developments in anti-severe acute respiratory syndrome coronavirus chemotherapy. Future Virol..

[63] Yilin Z., Yandong N., Faguang J. (2015). Role of angiotensin-converting enzyme (ACE) and ACE2 in a rat model of smoke inhalation induced acute respiratory distress syndrome. Burns.

[64] Kuba K., Imai Y., Rao S., Gao H., Guo F., Guan B., Huan Y., Yang P., Zhang Y., Deng W. (2005). A crucial role of angiotensin converting enzyme 2 (ACE2) in SARS coronavirus–induced lung injury. Nat. Med..

[65] Tolouian R., Vahed S.Z., Ghiyasvand S., Tolouian A., Ardalan M. (2020). COVID-19 interactions with angiotensin-converting enzyme 2 (ACE2) and the kinin system; looking at a potential treatment. J. Ren. Inj. Prev..

[66] Stewart C.A., Gay C.M., Ramkumar K., Cargill K.R., Cardnell R.J., Nilsson M.B., Heeke S., Park E.M., Kundu S.T., Diao L. (2020). SARS-CoV-2 infection induces EMT-like molecular changes, including ZEB1-mediated repression of the viral receptor ACE2, in lung cancer models. bioRxiv.

[67] Lin B., Ferguson C., White J.T., Wang S., Vessella R., True L.D., Hood L., Nelson P.S. (1999). Prostate-localized and androgen-regulated expression of the membrane-bound serine protease TMPRSS2. Cancer Res..

[68] Lucas J., True L., Hawley S., Matsumura M., Morrissey C., Vessella R., Nelson P. (2008). The androgen-regulated type II serine protease TMPRSS2 is differentially expressed and mislocalized in prostate adenocarcinoma. J. Pathol..

[69] Vaarala M.H., Porvari K.S., Kellokumpu S., Kyllönen A.P., Vihko P.T. (2001). Expression of transmembrane serine protease TMPRSS2 in mouse and human tissues. J. Pathol.

[70] Böttcher-Friebertshäuser E. (2018). Membrane-Anchored Serine Proteases: Host Cell Factors in Proteolytic Activation of Viral Glycoproteins. Activation of Viruses by Host Proteases.

[71] Böttcher E., Matrosovich T., Beyerle M., Klenk H.-D., Garten W., Matrosovich M. (2006). Proteolytic activation of influenza viruses by serine proteases TMPRSS2 and HAT from human airway epithelium. J. Virol..

[72] Böttcher-Friebertshäuser E., Freuer C., Sielaff F., Schmidt S., Eickmann M., Uhlendorff J., Steinmetzer T., Klenk H.D., Garten W. (2010). Cleavage of influenza virus hemagglutinin by airway proteases TMPRSS2 and HAT differs in subcellular localization and susceptibility to protease inhibitors. J. Virol..

[73] Chaipan C., Kobasa D., Bertram S., Glowacka I., Steffen I., Tsegaye T.S., Takeda M., Bugge T.H., Kim S., Park Y. (2009). Proteolytic activation of the 1918 influenza virus hemagglutinin. J. Virol..

[74] Kim T.S., Heinlein C., Hackman R.C., Nelson P.S. (2006). Phenotypic analysis of mice lacking the Tmprss2-encoded protease. Mol. Cell. Biol..

[75] Meyer M., Jaspers I. (2015). Respiratory protease/antiprotease balance determines susceptibility to viral infection and can be modified by nutritional antioxidants. Am. J. Physiol. Lung Cell. Mol. Physiol..

[76] Abboud R.T., Vimalanathan S. (2008). Pathogenesis of COPD. Part I. The role of protease-antiprotease imbalance in emphysema. Int. J. Tuberc. Lung Dis..

[77] Kersul A.L., Iglesias A., Ríos Á., Noguera A., Forteza A., Serra E., Agustí A., Cosío B.G. (2011). Molecular mechanisms of inflammation during exacerbations of chronic obstructive pulmonary disease. Arch. Bronconeumol..

[78] Sakai K., Ami Y., Tahara M., Kubota T., Anraku M., Abe M., Nakajima N., Sekizuka T., Shirato K., Suzaki Y. (2014). The Host Protease TMPRSS2 Plays a Major Role in In Vivo Replication of Emerging H7N9 and Seasonal Influenza Viruses. J. Virol..

[79] Shirato K., Kawase M., Matsuyama S. (2013). Middle East respiratory syndrome coronavirus infection mediated by the transmembrane serine protease TMPRSS2. J. Virol..

[80] Cheng Z., Zhou J., To K.K., Chu H., Li C., Wang D., Yang D., Zheng S., Hao K., Bossé Y. (2015). Identification of TMPRSS2 as a Susceptibility Gene for Severe 2009 Pandemic A(H1N1) Influenza and A(H7N9) Influenza. J. Infect. Dis..

[81] Williams J.V., Harris P.A., Tollefson S.J., Halburnt-Rush L.L., Pingsterhaus J.M., Edwards K.M., Wright P.F., Crowe J.E. (2004). Human metapneumovirus and lower respiratory tract disease in otherwise healthy infants and children. N. Engl. J. Med..

[82] Shirogane Y., Takeda M., Iwasaki M., Ishiguro N., Takeuchi H., Nakatsu Y., Tahara M., Kikuta H., Yanagi Y. (2008). Efficient Multiplication of Human Metapneumovirus in Vero Cells Expressing the Transmembrane Serine Protease TMPRSS2. J. Virol..

[83] Hatesuer B., Bertram S., Mehnert N., Bahgat M.M., Nelson P.S., Pöhlmann S., Schughart K. (2013). Tmprss2 is essential for influenza H1N1 virus pathogenesis in mice. PLoS Pathog..

[84] Fink S.L., Cookson B.T. (2005). Apoptosis, pyroptosis, and necrosis: Mechanistic description of dead and dying eukaryotic cells. Infect. Immun..

[85] Zhang L., Zhu F., Xie L., Wang C., Wang J., Chen R., Jia P., Guan H., Peng L., Chen Y. (2020). Clinical characteristics of COVID-19-infected cancer patients: A retrospective case study in three hospitals within Wuhan, China. Ann. Oncol..

[86] Chen I.Y., Moriyama M., Chang M.F., Ichinohe T. (2019). Severe Acute Respiratory Syndrome Coronavirus Viroporin 3a Activates the NLRP3 Inflammasome. Front. Microbiol..

[87] Zhang H., Zhou P., Wei Y., Yue H., Wang Y., Hu M., Zhang S., Cao T., Yang C., Li M. (2020). Histopathologic Changes and SARS-CoV-2 Immunostaining in the Lung of a Patient With COVID-19. Ann. Intern. Med..

[88] Xu Z., Shi L., Wang Y., Zhang J., Huang L., Zhang C., Liu S., Zhao P., Liu H., Zhu L. (2020). Pathological findings of COVID-19 associated with acute respiratory distress syndrome. Lancet Respir. Med..

[89] Tian S., Xiao S.Y. (2020). Pathology of 2019 Novel Coronavirus Pneumonia: A Dynamic Disease Process. J. Thorac. Oncol..

[90] Tay M.Z., Poh C.M., Rénia L., MacAry P.A., Ng L.F.P. (2020). The trinity of COVID-19: Immunity, inflammation and intervention. Nat. Rev. Immunol..

[91] Ruan Q., Yang K., Wang W., Jiang L., Song J. (2020). Correction to: Clinical predictors of mortality due to COVID-19 based on an analysis of data of 150 patients from Wuhan, China. Intensive Care Med..

[92] Zhou Y., Vedantham P., Lu K., Agudelo J., Carrion R., Nunneley J.W., Barnard D., Pöhlmann S., McKerrow J.H., Renslo A.R. (2015). Protease inhibitors targeting coronavirus and filovirus entry. Antivir. Res..

[93] Matsuyama S., Nao N., Shirato K., Kawase M., Saito S., Takayama I., Nagata N., Sekizuka T., Katoh H., Kato F. (2020). Enhanced isolation of SARS-CoV-2 by TMPRSS2-expressing cells. Proc. Natl. Acad. Sci. USA.

[94] Meng T., Cao H., Zhang H., Kang Z., Xu D., Gong H., Wang J., Li Z., Cui X., Xu H. (2020). The transmembrane serine protease inhibitors are potential antiviral drugs for 2019-nCoV targeting the insertion sequence-induced viral infectivity enhancement. bioRxiv.

[95] Li R., Qiao S., Zhang G. (2020). Analysis of angiotensin-converting enzyme 2 (ACE2) from different species sheds some light on cross-species receptor usage of a novel coronavirus 2019-nCoV. J. Infect..

[96] Simmons G., Gosalia D.N., Rennekamp A.J., Reeves J.D., Diamond S.L., Bates P. (2005). Inhibitors of cathepsin L prevent severe acute respiratory syndrome coronavirus entry. Proc. Natl. Acad. Sci. USA.

[97] Bertram S., Glowacka I., Müller M.A., Lavender H., Gnirss K., Nehlmeier I., Niemeyer D., He Y., Simmons G., Drosten C. (2011). Cleavage and Activation of the Severe Acute Respiratory Syndrome Coronavirus Spike Protein by Human Airway Trypsin-Like Protease. J. Virol..

[98] Lukassen S., Chua R.L., Trefzer T., Kahn N.C., Schneider M.A., Muley T., Winter H., Meister M., Veith C., Boots A.W. (2020). SARS-CoV-2 receptor ACE2 and TMPRSS2 are primarily expressed in bronchial transient secretory cells. EMBO J..

[99] Kong Q. (2020). Analysis the susceptibility of lung cancer patients to SARS-CoV-2 infection. Mol. Cancer.

[100] Pinto B.G., Oliveira A.E., Singh Y., Jimenez L., Goncalves A.N., Ogava R.L., Creighton R., Peron J.P., Nakaya H.I. (2020). ACE2 Expression is Increased in the Lungs of Patients with Comorbidities Associated with Severe COVID-19. medRxiv.

[101] Kim W.J., Lim J.H., Lee J.S., Lee S.-D., Kim J.H., Oh Y.-M. (2015). Comprehensive Analysis of Transcriptome Sequencing Data in the Lung Tissues of COPD Subjects. Int. J. Genom..

[102] Jia H.P., Look D.C., Tan P., Shi L., Hickey M., Gakhar L., Chappell M.C., Wohlford-Lenane C., McCray P.B. (2009). Ectodomain shedding of angiotensin converting enzyme 2 in human airway epithelia. Am. J. Physiol. Lung Cell. Mol. Physiol..

[103] Totura A.L., Whitmore A., Agnihothram S., Schäfer A., Katze M.G., Heise M.T., Baric R.S. (2015). Toll-Like Receptor 3 Signaling via TRIF Contributes to a Protective Innate Immune Response to Severe Acute Respiratory Syndrome Coronavirus Infection. mBio.

[104] Liang W., Guan W., Chen R., Wang W., Li J., Xu K., Li C., Ai Q., Lu W., Liang H. (2020). Cancer patients in SARS-CoV-2 infection: A nationwide analysis in China. Lancet Oncol..

[105] Hsueh W.A., Wyne K. (2011). Renin-angiotensin-aldosterone system in diabetes and hypertension. J. Clin. Hypertens..

[106] Lau S.T., Leung P.S. (2011). Role of the RAS in pancreatic cancer. Curr. Cancer Drug Targets.

[107] Geuna E., Lombardi P., Martinello R., Garino D., Bonzano A., Galizia D., Nuzzo A., Berchialla P., Becco P., Mangioni M. (2020). Treatment with Beta-Blockers and ACE-Inhibitors in Breast Cancer Patients Receiving Adjuvant Trastuzumab-Based Therapy and Developing Mild Cardiac Toxicity: A Prospective Study. Cancers.

[108] Babacan T., Balakan O., Kuzan T.Y., Sarici F., Koca E., Kertmen N., Petekkaya I., Altundag K. (2015). The effect of renin-angiotensin-system inhibition on survival and recurrence of N3+ breast cancer patients. J. Buon.

[109] Ino K., Shibata K., Yamamoto E., Kajiyama H., Nawa A., Mabuchi Y., Yagi S., Minami S., Tanizaki Y., Kobayashi A. (2011). Role of the renin-angiotensin system in gynecologic cancers. Curr. Cancer Drug Targets.

[110] Kuniyasu H. (2012). Multiple roles of angiotensin in colorectal cancer. World J. Clin. Oncol..

[111] De Paula G.A., Palmeira V., Ribeiro T., Costa L., de Sá R.K. (2020). ACE2/Angiotensin-(1-7)/Mas receptor axis in human cancer: Potential role for pediatric tumors. Curr. Drug Targets.

[112] Ramírez-Expósito M.J., Martínez-Martos J.M. (2018). Anti-Inflammatory and antitumor effects of hydroxytyrosol but not oleuropein on experimental glioma in vivo. A putative role for the renin-angiotensin system. Biomedicines.

[113] Tikellis C., Thomas M. (2012). Angiotensin-converting enzyme 2 (ACE2) is a key modulator of the renin angiotensin system in health and disease. Int. J. Pept..

[114] Passos-Silva D.G., Brandan E., Santos R.A.S. (2015). Angiotensins as therapeutic targets beyond heart disease. Trends Pharmacol. Sci..

[115] Rasha F., Ramalingam L., Gollahon L., Rahman R., Rahman S.M., Menikdiwela K., Moustaid-Moussa N. (2019). Mechanisms linking the renin-angiotensin system, obesity, and breast cancer. Endocr. Relat. Cancer.

[116] Qian Y.-R., Guo Y., Wan H.-Y., Fan L., Feng Y., Ni L., Xiang Y., Li Q.-Y. (2013). Angiotensin-converting enzyme 2 attenuates the metastasis of non-small cell lung cancer through inhibition of epithelial-mesenchymal transition. Oncol. Rep..

[117] Ni L., Feng Y., Wan H., Ma Q., Fan L., Qian Y., Li Q., Xiang Y., Gao B. (2012). Angiotensin-(1-7) inhibits the migration and invasion of A549 human lung adenocarcinoma cells through inactivation of the PI3K/Akt and MAPK signaling pathways. Oncol. Rep..

[118] Zhou L., Zhang R., Zhang L., Yao W., Li J., Yuan Y. (2011). Angiotensin-converting enzyme 2 acts as a potential molecular target for pancreatic cancer therapy. Cancer Lett..

[119] Yu C., Tang W., Wang Y., Shen Q., Wang B., Cai C., Meng X., Zou F. (2016). Downregulation of ACE2/Ang-(1–7)/Mas axis promotes breast cancer metastasis by enhancing store-operated calcium entry. Cancer Lett..

[120] e Silva A.C.S., Sampaio W.O. (2019). The Role of Angiotensin–(1-7) in Cancer. Angiotensin-(1-7).

[121] e Silva A.C.S., Teixeira M.M. (2016). ACE inhibition, ACE2 and angiotensin-(1–7) axis in kidney and cardiac inflammation and fibrosis. Pharmacol. Res..

[122] Gallagher P., Arter A., Deng G., Tallant E. (2014). Angiotensin-(1-7): A peptide hormone with anti-cancer activity. Curr. Med. Chem..

[123] Kuba K., Imai Y., Rao S., Jiang C., Penninger J.M. (2006). Lessons from SARS: Control of acute lung failure by the SARS receptor ACE2. J. Mol. Med..

[124] Delforce S.J., Lumbers E.R., de Meaultsart C.C., Wang Y., Proietto A., Otton G., Scurry J., Verrills N.M., Scott R.J., Pringle K.G. (2017). Expression of renin–angiotensin system (RAS) components in endometrial cancer. Endocr. Connect..

[125] Das U.N. (2020). Reply to: Bioactive Lipids and Coronavirus (COVID-19)-further Discussion. Arch. Med. Res..

[126] Antalis T.M., Bugge T.H., Wu Q., Di Cera E. (2011). Chapter 1-Membrane-Anchored Serine Proteases in Health and Disease. Progress in Molecular Biology and Translational Science.

[127] Tomlins S.A., Rhodes D.R., Perner S., Dhanasekaran S.M., Mehra R., Sun X.W., Varambally S., Cao X., Tchinda J., Kuefer R. (2005). Recurrent fusion of TMPRSS2 and ETS transcription factor genes in prostate cancer. Science.

[128] Yu J., Yu J., Mani R.-S., Cao Q., Brenner C.J., Cao X., Wang X., Wu L., Li J., Hu M. (2010). An Integrated Network of Androgen Receptor, Polycomb, and TMPRSS2-ERG Gene Fusions in Prostate Cancer Progression. Cancer Cell.

[129] Li Y., Sarkar F.H. (2002). Down-regulation of invasion and angiogenesis-related genes identified by cDNA microarray analysis of PC3 prostate cancer cells treated with genistein. Cancer Lett..

[130] Greenberg D.L., Mize G.J., Takayama T.K. (2003). Protease-activated receptor mediated RhoA signaling and cytoskeletal reorganization in LNCaP cells. Biochemistry.

[131] Cooper C.R., Chay C.H., Gendernalik J.D., Lee H.L., Bhatia J., Taichman R.S., McCauley L.K., Keller E.T., Pienta K.J. (2003). Stromal factors involved in prostate carcinoma metastasis to bone. Cancer.

[132] Vaarala M.H., Porvari K., Kyllönen A., Vihko P. (2000). Differentially expressed genes in two LNCaP prostate cancer cell lines reflecting changes during prostate cancer progression. Lab. Investig..

[133] Macfarlane S.R., Seatter M.J., Kanke T., Hunter G.D., Plevin R. (2001). Proteinase-activated receptors. Pharm. Rev..

[134] Bertram S., Glowacka I., Blazejewska P., Soilleux E., Allen P., Danisch S., Steffen I., Choi S.Y., Park Y., Schneider H. (2010). TMPRSS2 and TMPRSS4 facilitate trypsin-independent spread of influenza virus in Caco-2 cells. J. Virol..

[135] Yamaya M., Shimotai Y., Hatachi Y., Lusamba Kalonji N., Tando Y., Kitajima Y., Matsuo K., Kubo H., Nagatomi R., Hongo S. (2015). The serine protease inhibitor camostat inhibits influenza virus replication and cytokine production in primary cultures of human tracheal epithelial cells. Pulm. Pharm. Ther..

[136] Jung H., Lee K.P., Park S.J., Park J.H., Jang Y.S., Choi S.Y., Jung J.G., Jo K., Park D.Y., Yoon J.H. (2008). TMPRSS4 promotes invasion, migration and metastasis of human tumor cells by facilitating an epithelial-mesenchymal transition. Oncogene.

[137] Yasuoka S., Ohnishi T., Kawano S., Tsuchihashi S., Ogawara M., Masuda K., Yamaoka K., Takahashi M., Sano T. (1997). Purification, characterization, and localization of a novel trypsin-like protease found in the human airway. Am. J. Respir. Cell Mol. Biol..

[138] Yamaoka K., Masuda K., Ogawa H., Takagi K., Umemoto N., Yasuoka S. (1998). Cloning and characterization of the cDNA for human airway trypsin-like protease. J. Biol. Chem..

[139] Guo C.C., Dancer J.Y., Wang Y., Aparicio A., Navone N.M., Troncoso P., Czerniak B.A. (2011). TMPRSS2-ERG gene fusion in small cell carcinoma of the prostate. Hum. Pathol..

[140] Passaro A., Peters S., Mok T.S.K., Attili I., Mitsudomi T., de Marinis F. (2020). Testing for COVID-19 in lung cancer patients. Ann. Oncol..

[141] Zhao Z., Bai H., Duan J., Wang J. (2020). Recommendations of individualized medical treatment and common adverse events management for lung cancer patients during the outbreak of COVID-19 epidemic. Thorac. Cancer.

[142] Chinese T.S., Group L.C.S., Association C.M., Collaboration C.R.O. (2020). Expert recommendations on the management of patients with advanced non-small cell lung cancer during epidemic of COVID-19 (Trial version). Zhonghua Jie He He Hu Xi Za Zhi = Zhonghua Jiehe He Huxi Zazhi = Chin. J. Tuberc. Respir. Dis..

[143] Hung Y.-H., Hsieh W.-Y., Hsieh J.-S., Liu C., Tsai C.-H., Lu L.-C., Huang C.-Y., Wu C.-L., Lin C.-S. (2016). Alternative roles of STAT3 and MAPK signaling pathways in the MMPs activation and progression of lung injury induced by cigarette smoke exposure in ACE2 knockout mice. Int. J. Biol. Sci..

[144] Rubinfeld H., Seger R. (2005). The ERK cascade: A prototype of MAPK signaling. Mol. Biotechnol..

[145] Bang Y.J., Kwon J.H., Kang S.H., Kim J.W., Yang Y.C. (1998). Increased MAPK activity and MKP-1 overexpression in human gastric adenocarcinoma. Biochem. Biophys. Res. Commun..

[146] Lefloch R., Pouysségur J., Lenormand P. (2009). Total ERK1/2 activity regulates cell proliferation. Cell Cycle.

[147] Khatlani T.S., Wislez M., Sun M., Srinivas H., Iwanaga K., Ma L., Hanna A.E., Liu D., Girard L., Kim Y.H. (2007). c-Jun N-terminal kinase is activated in non-small-cell lung cancer and promotes neoplastic transformation in human bronchial epithelial cells. Oncogene.

[148] Mizutani T., Fukushi S., Murakami M., Hirano T., Saijo M., Kurane I., Morikawa S. (2004). Tyrosine dephosphorylation of STAT3 in SARS coronavirus-infected Vero E6 cells. FEBS Lett..

[149] Mizutani T., Fukushi S., Saijo M., Kurane I., Morikawa S. (2004). Phosphorylation of p38 MAPK and its downstream targets in SARS coronavirus-infected cells. Biochem. Biophys. Res. Commun..

[150] Mizutani T., Fukushi S., Saijo M., Kurane I., Morikawa S. (2005). JNK and PI3k/Akt signaling pathways are required for establishing persistent SARS-CoV infection in Vero E6 cells. Biochim. Biophys. Acta.

[151] Greenberg A.K., Basu S., Hu J., Yie T.-a., Tchou-Wong K.M., Rom W.N., Lee T.C. (2002). Selective p38 Activation in Human Non–Small Cell Lung Cancer. Am. J. Respir. Cell Mol. Biol..

[152] Chen I.Y., Chang S.C., Wu H.Y., Yu T.C., Wei W.C., Lin S., Chien C.L., Chang M.F. (2010). Upregulation of the chemokine (C-C motif) ligand 2 via a severe acute respiratory syndrome coronavirus spike-ACE2 signaling pathway. J. Virol..

[153] Lim Y.X., Ng Y.L., Tam J.P., Liu D.X. (2016). Human Coronaviruses: A Review of Virus-Host Interactions. Diseases.

[154] Liu M., Yang Y., Gu C., Yue Y., Wu K.K., Wu J., Zhu Y. (2007). Spike protein of SARS-CoV stimulates cyclooxygenase-2 expression via both calcium-dependent and calciumin-dependent protein kinase C pathways. FASEB J..

[155] Fung T.S., Liao Y., Liu D.X. (2014). The endoplasmic reticulum stress sensor IRE1α protects cells from apoptosis induced by the coronavirus infectious bronchitis virus. J. Virol..

[156] Fung T.S., Liu D.X. (2017). Activation of the c-Jun NH(2)-terminal kinase pathway by coronavirus infectious bronchitis virus promotes apoptosis independently of c-Jun. Cell Death Dis..

[157] Mehta P., McAuley D.F., Brown M., Sanchez E., Tattersall R.S., Manson J.J. (2020). COVID-19: Consider cytokine storm syndromes and immunosuppression. Lancet.

[158] Kawase M., Shirato K., van der Hoek L., Taguchi F., Matsuyama S. (2012). Simultaneous treatment of human bronchial epithelial cells with serine and cysteine protease inhibitors prevents severe acute respiratory syndrome coronavirus entry. J. Virol..

[159] Gurwitz D. (2020). Angiotensin receptor blockers as tentative SARS-CoV-2 therapeutics. Drug Dev. Res..

[160] Touyz R.M., Li H., Delles C. (2020). ACE2 the Janus-faced protein–from cardiovascular protection to severe acute respiratory syndrome-coronavirus and COVID-19. Clin. Sci..

[161] Kuster G.M., Pfister O., Burkard T., Zhou Q., Twerenbold R., Haaf P., Widmer A.F., Osswald S. (2020). SARS-CoV2: Should inhibitors of the renin–angiotensin system be withdrawn in patients with COVID-19?. Eur. Heart J..

[162] McKay R.R., Rodriguez G.E., Lin X., Kaymakcalan M.D., Hamnvik O.-P.R., Sabbisetti V.S., Bhatt R.S., Simantov R., Choueiri T.K. (2015). Angiotensin system inhibitors and survival outcomes in patients with metastatic renal cell carcinoma. Clin. Cancer Res..

[163] Neo J.H., Ager E.I., Angus P.W., Zhu J., Herath C.B., Christophi C. (2010). Changes in the renin angiotensin system during the development of colorectal cancer liver metastases. BMC Cancer.

[164] Zhao Y., Chen X., Cai L., Yang Y., Sui G., Fu S. (2010). Angiotensin II/angiotensin II type I receptor (AT1R) signaling promotes MCF-7 breast cancer cells survival via PI3-kinase/Akt pathway. J. Cell. Physiol..

[165] Aydiner A., Ciftci R., Sen F. (2015). Renin-Angiotensin system blockers may prolong survival of metastatic non-small cell lung cancer patients receiving erlotinib. Medicine.

[166] Mendoza-Torres E., Oyarzún A., Mondaca-Ruff D., Azocar A., Castro P.F., Jalil J.E., Chiong M., Lavandero S., Ocaranza M.P. (2015). ACE2 and vasoactive peptides: Novel players in cardiovascular/renal remodeling and hypertension. Ther. Adv. Cardiovasc. Dis..

[167] Wong S.K., Li W., Moore M.J., Choe H., Farzan M. (2004). A 193-amino acid fragment of the SARS coronavirus S protein efficiently binds angiotensin-converting enzyme 2. J. Biol. Chem..

[168] Wang X., Mathieu M., Brezski R.J. (2018). IgG Fc engineering to modulate antibody effector functions. Protein Cell.

[169] Li W., Moore M.J., Vasilieva N., Sui J., Wong S.K., Berne M.A., Somasundaran M., Sullivan J.L., Luzuriaga K., Greenough T.C. (2003). Angiotensin-converting enzyme 2 is a functional receptor for the SARS coronavirus. Nature.

[170] Kruse R.L. (2020). Therapeutic strategies in an outbreak scenario to treat the novel coronavirus originating in Wuhan, China. F1000Research.

[171] Moore M.J., Dorfman T., Li W., Wong S.K., Li Y., Kuhn J.H., Coderre J., Vasilieva N., Han Z., Greenough T.C. (2004). Retroviruses pseudotyped with the severe acute respiratory syndrome coronavirus spike protein efficiently infect cells expressing angiotensin-converting enzyme 2. J. Virol..

[172] Abe M., Tahara M., Sakai K., Yamaguchi H., Kanou K., Shirato K., Kawase M., Noda M., Kimura H., Matsuyama S. (2013). TMPRSS2 is an activating protease for respiratory parainfluenza viruses. J. Virol..

[173] Shin W.J., Seong B.L. (2017). Type II transmembrane serine proteases as potential target for anti-influenza drug discovery. Expert Opin. Drug Discov..

[174] Yamamoto M., Matsuyama S., Li X., Takeda M., Kawaguchi Y., Inoue J.-i., Matsuda Z. (2016). Identification of Nafamostat as a Potent Inhibitor of Middle East Respiratory Syndrome Coronavirus S Protein-Mediated Membrane Fusion Using the Split-Protein-Based Cell-Cell Fusion Assay. Antimicrob. Agents Chemother..

[175] Hoffmann M., Kleine-Weber H., Schroeder S., Krüger N., Herrler T., Erichsen S., Schiergens T.S., Herrler G., Wu N.H., Nitsche A. (2020). SARS-CoV-2 Cell Entry Depends on ACE2 and TMPRSS2 and Is Blocked by a Clinically Proven Protease Inhibitor. Cell.

[176] Böttcher-Friebertshäuser E., Lu Y., Meyer D., Sielaff F., Steinmetzer T., Klenk H.D., Garten W. (2012). Hemagglutinin activating host cell proteases provide promising drug targets for the treatment of influenza A and B virus infections. Vaccine.

[177] Kim H., Datta A., Talwar S., Saleem S.N., Mondal D., Abdel-Mageed A.B. (2016). Estradiol-ERβ2 signaling axis confers growth and migration of CRPC cells through TMPRSS2-ETV5 gene fusion. Oncotarget.

[178] Li Y., Kong D., Wang Z., Ahmad A., Bao B., Padhye S., Sarkar F.H. (2011). Inactivation of AR/TMPRSS2-ERG/Wnt signaling networks attenuates the aggressive behavior of prostate cancer cells. Cancer Prev. Res..

[179] Semaan L., Mander N., Cher M.L., Chinni S.R. (2019). TMPRSS2-ERG fusions confer efficacy of enzalutamide in an in vivo bone tumor growth model. BMC Cancer.

[180] Ateeq B., Vellaichamy A., Tomlins S.A., Wang R., Cao Q., Lonigro R.J., Pienta K.J., Varambally S. (2012). Role of dutasteride in pre-clinical ETS fusion-positive prostate cancer models. Prostate.

[181] Knuuttila M., Mehmood A., Mäki-Jouppila J., Ryberg H., Taimen P., Knaapila J., Ettala O., Boström P.J., Ohlsson C., Venäläinen M.S. (2018). Intratumoral androgen levels are linked to TMPRSS2-ERG fusion in prostate cancer. Endocr. Relat. Cancer.

[182] Channappanavar R., Fett C., Mack M., Ten Eyck P.P., Meyerholz D.K., Perlman S. (2017). Sex-Based Differences in Susceptibility to Severe Acute Respiratory Syndrome Coronavirus Infection. J. Immunol..

[183] Dong E., Du H., Gardner L. (2020). An interactive web-based dashboard to track COVID-19 in real time. Lancet Infect. Dis..

